# Vehicle ergonomics contributing to a diabetic foot ulcer

**DOI:** 10.1186/s40842-019-0089-4

**Published:** 2019-11-07

**Authors:** Christine Jarocki, Brian M. Schmidt, Crystal Murray Holmes

**Affiliations:** Department of Internal Medicine, Division of Metabolism, Endocrinology, and Diabetes, University of Michigan Hospital and Health System, Domino’s Farms Lobby G, Suite 1500, 24 Frank Lloyd Wright Drive, Ann Arbor, MI 48106 USA

**Keywords:** Diabetic peripheral neuropathy, Diabetic foot, Diabetic foot ulcer, Driving simulator, Diabetes mellitus, Pressure

## Abstract

**Background:**

Diabetes mellitus continues to be a rising concern in the United States. It affects an estimated 9.4% of the population and approximately 1.5 million Americans are diagnosed annually. Approximately 85% of diabetic foot ulcers are associated with diabetic peripheral neuropathy and an infected diabetic foot ulcer is often the first sign of diabetes. There are countless studies within the literature that investigate how insensate feet and the manifestation of a foot ulcer further decrease quality of life and increase risk for mortality. Literature focuses on gait and kinematics that contribute to the formation of a diabetic foot ulcer. While pressure and shear forces are etiologic factors that may lead to the formation of diabetic foot ulcers, the position of the foot while driving an automobile has been ignored as a possible risk factor.

**Case presentation:**

The clinical case will describe the events of healing a neuropathic diabetic foot ulcer beyond the standard of care treatment plan. It is one of the first case reports to describe vehicle ergonomics as an etiologic factor contributing to a diabetic foot ulcer. Once the patient becomes aware of the unnecessary source of pressure, education and care is provided to manage this likely source of daily pressure to the neuropathic foot.

**Conclusion:**

The article emphasizes the importance of a complete assessment, including nontraditional factors, which may lead to diabetic complications.

## Background

As the literature reports, diabetes mellitus (DM) continues to be a rising concern in the United States. Approximately 9.4% of the population, or 30.3 million people, are treated for this disease [1]. Estimations suggest that 1.5 million Americans are diagnosed yearly [1]. Approximately 15–25% of the DM population develop a diabetic foot ulcer (DFU) [2]. This may represent an underestimation given DFU do not occur with simple prediction patterns [3]. Thus, prevention of a DFU is difficult. The occurrence of a DFU places an increased mortality risk compared to a person with DM that does not have a DFU [4]. Diabetic peripheral neuropathy (DPN) is associated with approximately 85% of DFU [5–8]. Diabetes continues to be the primary cause for polyneuropathy in the United States [9]. Diabetic peripheral neuropathy is prevalent with patients of DM and prediabetes [8]. Further, prevalence of the complication increases with age and duration of DM [7].

Diabetes has been associated with three different types of DPN: sensory neuropathy, motor neuropathy, autonomic neuropathy [3]. Sensory neuropathy is the most common type of neuropathy associated with DM [10, 11]. The loss of protective sensation, temperature discrimination, and proprioception prevents biofeedback when there is increased stress on the foot [8]. Patients become unaware of incidental trauma and pain [12]. Peripheral sensory neuropathy is further coupled with motor neuropathy and can progress to decreased muscle innervation as a late finding. The mechanism of which motor neuropathy affects a person with DM is still not fully understood. Patients with type 2 diabetes mellitus [T2DM] may have decreased strength in the proximal and distal musculature of the lower extremities, and there is an increase in quantity of intramuscular non-contractile tissue [13]. The literature does not support motor neuropathy as a direct etiologic factor for biomechanical pedal deformities; however, there are atrophic effects on the proximal chain that could affect the anatomic position and function of the foot [14–16]. Lastly, autonomic neuropathy creates sweat gland dysfunction. When the sweat glands are unable to modify plantar foot temperatures, this increase can contribute to tissue breakdown [17, 18].

Diabetic lower extremity complications and driving automobiles is a paucity in research. One report series proposed that DM is associated with increased braking response time [19] and DPN exacerbates this delay [20]. Another report looked at accelerator pedal control in DM and DPN, and found an increase in foot pedal application error through ankle repositioning [21]. This research questions driver safety, but there appears to be little evidence that justifies permanent driving restrictions. Considering that state legislature determines driving regulations, it may be difficult to come to a national agreement [22].

Additional risks of driving automobiles are still largely ignored. These would include vehicle-driver relations, or vehicle ergonomics. The following clinical case will highlight an example of vehicle ergonomics contributing to a DFU.

## Case presentation

A 76-year-old obese man was admitted for inpatient management regarding a non-healing DFU. This ulcer was located at the plantar posterior right heel. His previous treatment involved regular care at a comprehensive wound care clinic, in which he returned for frequent debridement and assessment of topical wound care for more than 4 months. Hindering factors included lack of wound care assistance at home and inadequate offloading.

His past medical history included T2DM, DPN, chronic venous insufficiency with lymphedema, in addition to other co-morbidities. His DM was followed by a general medicine practitioner, using metformin and lifestyle modifications. His A1c value during the hospital admission was 5.6%. He had severe DPN. On exam there was absent protective sensation distal to the patella as demonstrated with the Semmes-Weinstein 10-g monofilament. Vibration was absent distal to the ankle joint according to the 128 Hz Tuning Fork. Manual manipulation indicated absent proprioception distal to the ankle joint. Muscle strength was found to be 5/5 with dorsiflexion and plantarflexion, and 4/5 with forced eversion and inversion. His distal lower extremity pulses were palpable.

**Fig. 1:**
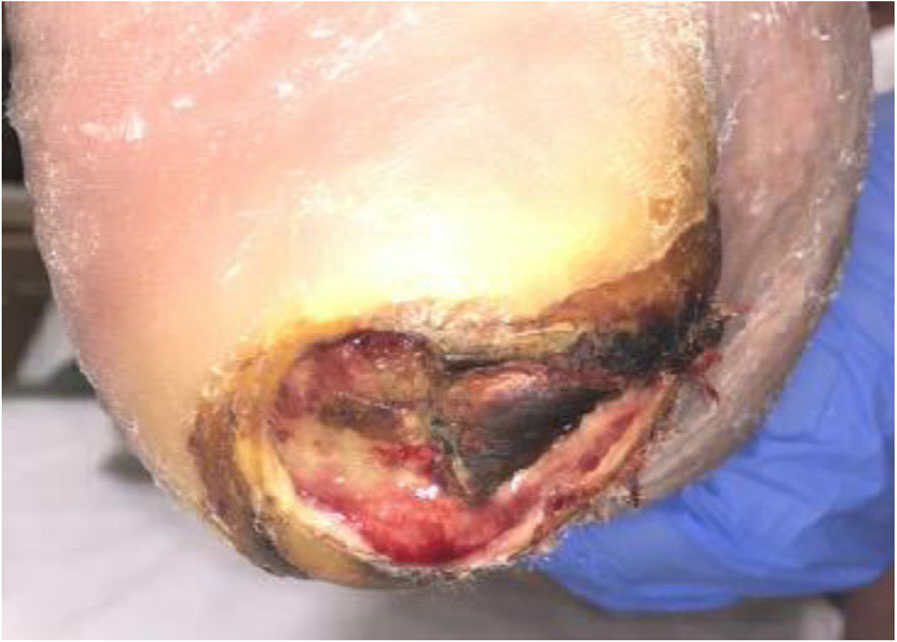
DFU at plantar posterior right heel

At the plantar posterior right heel there was an ovoid shaped ulcer that probed to the calcaneus [Fig. 1]. It measured approximately 5.5 × 2.5 cm [13.75cm^2^]. There were no acute clinical signs of infection. A magnetic resonance imaging was equivocal for “early osteomyelitis” versus reactive edema. The bone culture obtained was positive, and infectious disease treated the bacterial infection with 6 weeks of intravenous antibiotics. The patient followed with podiatry for wound care.

**Fig. 2:**
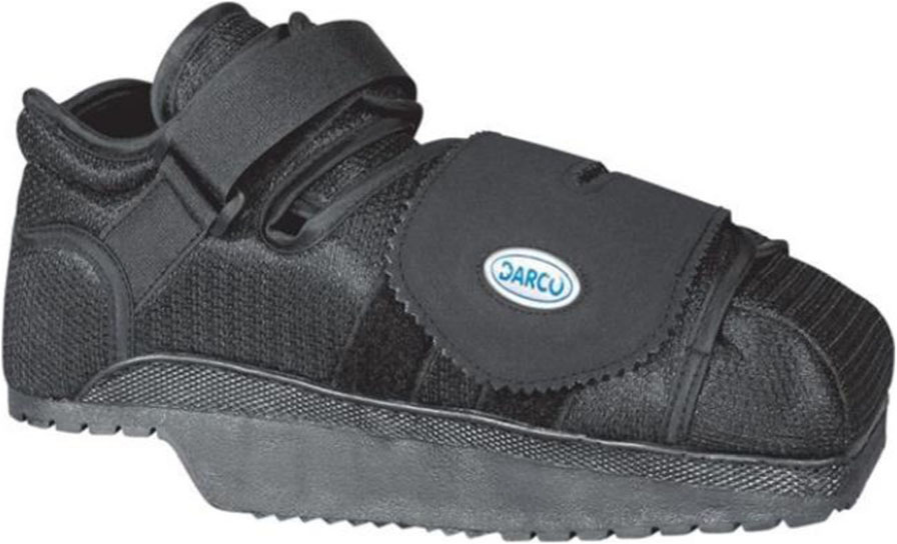
DARCO® HeelWedge™ shoe

**Fig. 3:**
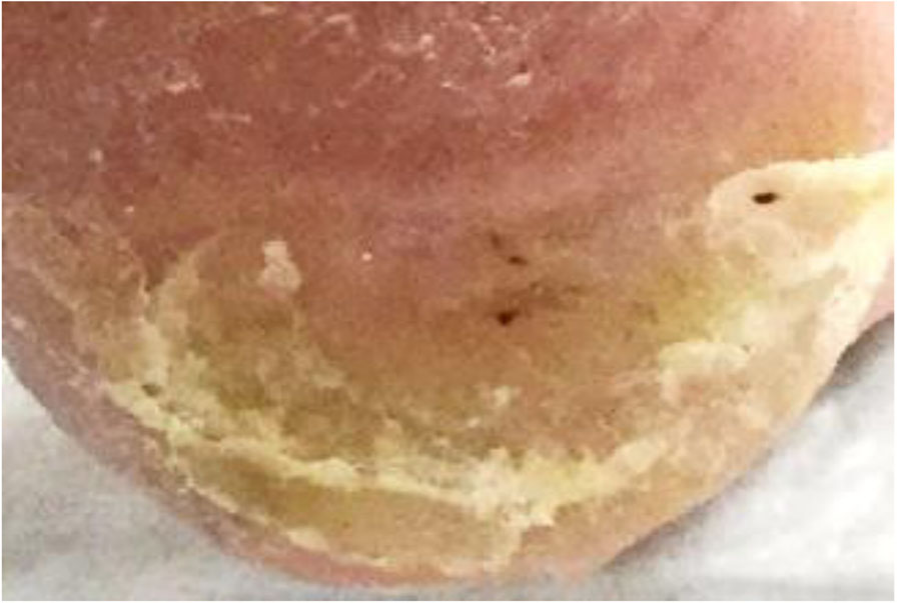
Healed DFU at plantar posterior right heel

Daily wound care consisted of a silver alginate dressing. Offloading was emphasized with this new treatment plan. He was offloaded with a DARCO® HeelWedge™ shoe (Huntington, West Virginia) [Fig. 2] and the assistance of a walker. The patient admitted to being the principal household driver and he drove frequently for recreation, boasting of driving millions of miles over his lifetime. Given the ulcer was located on the right lower extremity at an area of pressure with automobile pedal usage, temporary driving restrictions were given. He was advised not to drive during the healing process. In 6 months the ulcer closed [Fig. 3]. He was to remain in his offloading shoe for tissue remodeling and advised to monitor his foot for pressure throughout the day with different activities.

**Fig. 4:**
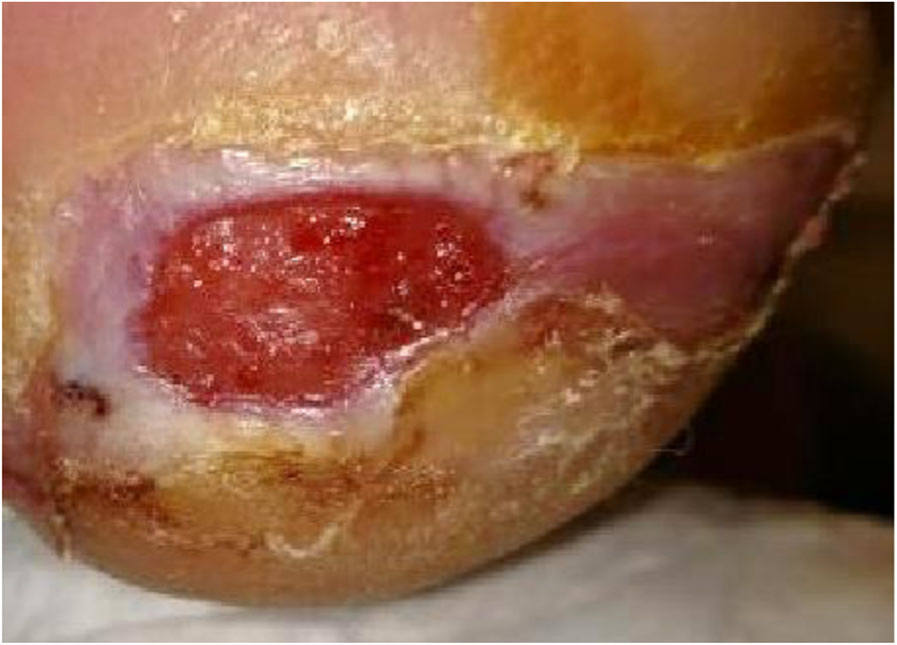
Re-ulcerated DFU at plantar posterior right heel

Approximately 1 month later, the patient called for an urgent appointment when his ulcer reoccurred [Fig. 4]. He admitted to driving more than 14 h over a couple days. He felt driving played a significant factor in his re-ulceration because of how his foot rested when using the foot pedals. He confirmed prior to his travels, there was no ulcer. He further confirmed that there was no change in his pattern of wearing his offloading shoe and using his walker. The patient returned to his prior treatment plan and proceeded to heal.

## Discussion

The clinical case draws attention to a daily life element that may be overlooked as a contributing source of pressure. When considering this patient, his ulcer was on his right heel at the plantar posterior junction. This area is the foot resting point for the accelerator and brake pedal of the right lower extremity and may be the dead pedal resting point of the left lower extremity. It is reasonable to assume that rigorous driving over 2 days could have contributed greatly to his re-ulceration, but does daily driving have a hindering effect on tissue healing? There has been one case study that contributed callus formation as a result from driving. The active driver developed callus to his left lateral heel due to inappropriate foot position that was influenced by his vehicle and seat geometry [23].

According to the National Highway Traffic Safety Administration, the HYBRID III Fiftieth Percentile crash test dummy is the most widely used dummy in frontal crash and automotive safety restraint testing. This device represents the average adult male, with weight of 78.2 kg (kg). The design further segments body weight into limbs. A lower leg and foot is contributed to 7.25% of the total body weight [24]. Type 2 Diabetes Mellitus has a positive correlation with body mass index [BMI] [1, 25]. There is potential that a person with a higher BMI, could have a larger lower limb. Thus, a greater force would be supported by the heel over a similar amount of area. The patient of the clinical case weighed 107 kg during his treatment plan. To calculate the actual pressure exerted from supporting his limb, the specific anthropometric patterns unique to him, within his vehicle, would need to be considered. A simple calculation that shows the potential increased amount of pressure uses ratios. The average male at 78.2 kg has a total mass of 5.7 kg to his lower leg. The presented patient, at 107 kg, would have a total mass of 7.8 kg to his lower leg. This is a 37% increase in distributed mass compared to the average male. Upon a search of databases including PubMed, NCBI, Google Scholar and SAE, no further literature or case reports are available relating to pressure exerted to the heel with driving automobiles.

Research on Diabetic peripheral neuropathy and driving automobiles is limited in the literature. One report series creates an association between DM and DPN with increased braking response time [19, 20], and another report associates DM and DPN with altered speed of strength generation and increased ankle reposition error [21]. These investigations question the safety for the driver, and all users of the road. None of these research designs looked at driver foot patterns as it relates to tissue pressure. Further, these were performed using driving simulators. Many reports show the benefits and validity of using such technology for brake response times, lateral positions, inattention, etc. [26–30]. However, the technology and architecture of a driving simulator system varies. One oversight of a driving simulator design is the difference with real life vehicle ergonomics. Human body posture and foot position fluctuate greatly when driving an automobile. Contributing factors include the individual height or shoe size in relation to the type of motor vehicle [31, 32]. It has been suggested that with a longer shoe or taller stature, a foot transfers by pivoting off the heel, while a shorter shoe or stature, a foot is more likely to move with lifting off the pedals [31, 33]. It has been further construed that foot pedal patterns are not necessarily habitual behavior, but rather anthropometrically dependent [31]. If a person is not able to feel the environment, it would be difficult to interact with it. A person with DPN may not know if foot placement or posture is contributing excessive pressure to their foot and they may be unaware with how their body is interacting with the vehicle.

It is well established that DPN affects gait [8, 34, 35]. It would be reasonable to accept the idea that DPN also alters the real life vehicle ergonomics of the driver. Bad vehicle ergonomics is known to result in repetitive motion injuries, soft tissue disorders, and fatigue in the general population [32]. We theorize that patients with DPN have different foot pedal patterns and this difference is not accounted for in studies with driving simulators. There is no standardized design for pedal placement within automobiles, but the accelerator, brake, and clutch pedal are some of the most frequently used vehicle parts [32]. If there is a unique pattern to this population group, there may be a vehicle design that would be safer and more streamlined for a person with DM and DPN.

This would be an important discovery. In certain regions, the ability to drive an automobile is considered a necessity. The National Highway Traffic Safety Administration acknowledges that a driver license symbolizes independence and freedom. Previous work suggests that removing this ability in older drivers is associated with increased depression and accelerated decline of cognitive function [36–40]. Given there is a known association with DM and depression and cognitive decline [41, 42], careful consideration is necessary when discussing driving activity with these patients. Driving discussions can be a difficult conversation to have; however, applying the understanding with how patients interact in their vehicles would be an important first step in patient education.

## Conclusion

In conclusion, this clinical case demonstrates the importance of considering nontraditional factors, such as vehicle ergonomics, as part of a complete assessment in the development of a DFU.

## Data Availability

Not applicable

## Abbreviations

BMI: Body Mass Index
DFU: Diabetic Foot Ulcer.
DM: Diabetes Mellitus.
DPN: Diabetic Peripheral Neuropathy
kg: Kilogram
T2DM: Type 2 Diabetes Mellitus

## References

[1] National Diabetes Statistics Report. 2017 [cited 17 September 2019]. Available from: https://www.cdc.gov/diabetes/pdfs/data/statistics/national-diabetes-statistics-report.pdf

[2] Singh N, Armstrong DG, Lipsky BA (2005). Preventing foot ulcers in patients with diabetes. Jama.

[3] Armstrong DG, Boulton AJM, Bus SA (2017). Diabetic foot ulcers and their recurrence. N Engl J Med.

[4] Boyko EJ, Ahroni JH, Smith DG, Davignon D (1996). Increased mortality associated with diabetic foot ulcer. Diabetic Med.

[5] Boulton AJ (1996). The pathogenesis of diabetic foot problems: an overview. Diabetic Med.

[6] Boulton AJ (2013). The pathway to foot ulceration in diabetes. Med Clin North Am.

[7] Boulton AJ, Malik RA, Arezzo JC, Sosenko JM (2004). Diabetic somatic neuropathies. Diabetes Care.

[8] Pop-Busui R, Boulton AJ, Feldman EL, Bril V, Freeman R, Malik RA (2017). Diabetic neuropathy: a position statement by the American Diabetes Association. Diabetes Care.

[9] National Institute of Neurological Disorders and Stroke: Peripheral Neuropathy Fact Sheet. 2019 [cited 17 September 2019]. Available from: https://www.ninds.nih.gov/Disorders/Patient-Caregiver-Education/Fact-Sheets/Peripheral-Neuropathy-Fact-Sheet

[10] Albers JW, Pop-Busui R (2014). Diabetic neuropathy: mechanisms, emerging treatments, and subtypes. Curr Neurol Neurosci Rep.

[11] Bansal V, Kalita J, Misra UK (2006). Diabetic neuropathy. Postgrad Med J.

[12] Boulton AJ (2012). Diabetic neuropathy: is pain God's greatest gift to mankind?. Semin Vasc Surg.

[13] Almurdhi MM, Reeves ND, Bowling FL, Boulton AJ, Jeziorska M, Malik RA (2016). Reduced lower-limb muscle strength and volume in patients with type 2 diabetes in relation to neuropathy, intramuscular fat, and vitamin D levels. Diabetes Care.

[14] Bus SA, Maas M, Michels RP, Levi M (2009). Role of intrinsic muscle atrophy in the etiology of claw toe deformity in diabetic neuropathy may not be as straightforward as widely believed. Diabetes Care.

[15] Allan J, Munro W, Figgins E (2016). Foot deformities within the diabetic foot and their influence on biomechanics: a review of the literature. Prosthetics Orthot Int.

[16] Andersen H (2012). Motor dysfunction in diabetes. Diabetes Metab Res Rev.

[17] Yavuz M, Ersen A, Hartos J, Lavery LA, Wukich DK, Hirschman GB, et al. Temperature as a causative factor in diabetic foot ulceration: a call to revisit ulcer Pathomechanics. J Am Podiatr Med Assoc. 2018. 10.7547/17-131 30427732

[18] Schmidt BM, Allison S, Wrobel JS. Describing normative foot temperatures in patients with diabetes-related peripheral neuropathy. J Diabetes Sci Technol. 2019:1932296819864664. 10.1177/1932296819864664 PMC7189153 31315460

[19] Meyr AJ SKE (2017). Diabetic Driving Studies – Part 1: Brake Response Time in Diabetic Drivers With Lower Extremity Neuropathy. J Foot Ankle Surg.

[20] Sansosti SKE, AJ LEM (2017). Diabetic Driving Studies – Part 2: A Comparison of Brake Response Time Between Drivers With Diabetes With and Without Lower Extremity Sensorimotor Neuropathy. J Foot Ankle Surg.

[21] Perazzolo M, Reeves ND, Bowling FL, Boulton AJM, Raffi M, Marple-Horvat DE. Altered accelerator pedal control in a driving simulator in people with diabetic peripheral neuropathy. Diabetic Med. 2019. 10.1111/dme.13957 PMC7004113 30924960

[22] Sansosti LE, Greene T, Hasenstein T, Berger M, Meyr AJ (2017). U.S. State Driving Regulations Relevant to Foot and Ankle Surgeons. J Foot Ankle Surg.

[23] Rajput B, Abboud RJ (2007). The inadequate effect of automobile seating on foot posture and callus development. Ergonomics..

[24] National Highway Traffic Safety Administration:HYBRID III Fiftieth Percentile Male. 2014 [cited 16 September 2019]. Available from: http://www.nhtsa.gov.edgesuite-staging.net/Research/Hybrid+III+50th+Percentile+Male/

[25] American Diabetes Association. Obesity Management for the Treatment of Type 2 Diabetes: Standards of Medical Care in Diabetes-2019. Diabetes care. 2019;42(Suppl 1):S81-s9. 10.2337/dc19-S008 30559234

[26] Blana, E. Driving simulator validation studies: a literature review. University of Leeds 1996.

[27] Casutt G, Martin M, Keller M, Jancke L. The relation between performance in on-road driving, cognitive screening and driving simulator in older healthy drivers. Transp Res F. 2013.

[28] Hoskins AH, El-Gindy M. Technical report: Literature survey on driving simulator validation studies. International Journal of Heavy Vehicle Systems. 2006. [2], 241–252.

[29] Meuleners LB, Fraser M. A validation study of driving errors using a driving simulator. Transp Res F. 2015.

[30] Sahami S, Sayed S. How drivers adapt to drive in driving simulator, and what is the impact of practice scenario on the research? Transportation Research Part F. 2013. [16]:41–52.

[31] Yubin Xi JB, Paul Venhovens, Patrick Rosopa, John DesJardins, Shayne McConomy, Leah Belle, Nathalie Drouin, Sarah Hennessy, Kevin Kopera, Jeremy McKee, Stephanie Tanner, Constance, Truesdail KL, Loren Staplin. Understanding the Automotive Pedal Usage and Foot Movement Characteristics of Older Drivers. SAE Technical Paper. 2018.

[32] Sahil Garg SB, Shashank Gupta. Analysis of Automotive Control Pedals Ergonomics through Mathematical Modelling Based on Human Anthropometry. SAE Technical Paper. 2017.

[33] Crandall JR, Martin P G, Bass C R, Pilkey W D, Dischinger, P C Burgess A R, O'Quinn T D, Schmidhauser C B. Foot and Ankle Injury: The Role of Driver Antrhopometry, Footwear, and Pedal Controls. Annual Proceedings of the Association for the Advancement of Automotive Medicine 1996.

[34] Morrison S., Colberg S.R., Parson H.K., Vinik A.I. (2012). Relation between risk of falling and postural sway complexity in diabetes. Gait & Posture.

[35] Brown Steven J., Handsaker Joseph C., Bowling Frank L., Boulton Andrew J.M., Reeves Neil D. (2015). Diabetic Peripheral Neuropathy Compromises Balance During Daily Activities. Diabetes Care.

[36] Chihuri S, Mielenz TJ, DiMaggio CJ, Betz ME, DiGuiseppi C, Jones VC (2016). Driving cessation and health outcomes in older adults. J Am Geriatr Soc.

[37] Choi M, Lohman MC, Mezuk B (2014). Trajectories of cognitive decline by driving mobility: evidence from the health and retirement study. Int J Geriatr Psychiatry.

[38] Fonda SJ, Wallace RB, Herzog AR (2001). Changes in driving patterns and worsening depressive symptoms among older adults. J Gerontol Ser B Psychol Sci Soc Sci.

[39] Marottoli RA, Mendes de Leon CF, Glass TA, Williams CS, Cooney LM, Berkman LF (1997). Driving cessation and increased depressive symptoms: prospective evidence from the New Haven EPESE. Established Populations for Epidemiologic Studies of the Elderly. J Am Geriatr Soc.

[40] Ragland DR, Satariano WA, MacLeod KE (2005). Driving cessation and increased depressive symptoms. J Gerontol A Biol Sci Med Sci.

[41] Badescu SV, Tataru C, Kobylinska L, Georgescu EL, Zahiu DM, Zagrean AM (2016). The association between diabetes mellitus and depression. J Med Life.

[42] Natovich R, Kushnir T, Harman-Boehm I, Margalit D, Siev-Ner I, Tsalichin D, et al. Cognitive dysfunction: part and parcel of the diabetic foot. Diabetes Care. 10.2337/dc15-2838 27208339

